# Paralog-specific miRNA regulation uncouples ASER gene repression from the canonical *die-1* / *cog-1* switch

**DOI:** 10.17912/micropub.biology.002255

**Published:** 2026-07-22

**Authors:** Lara C. Khalaf, Dylan L. Castro, Sayeda R. Qubadi, Ray L. Hong

**Affiliations:** 1 Biology Department, California State University, Northridge, Northridge, CA, United States

## Abstract

The *Pristionchus pacificus* ASER-specific *Ppa-gcy-22 .3* and *Ppa-gcy-22 .5* paralogs differ in their regulatory dependencies despite sharing an upstream terminal selector machinery. *P. pacificus gcy-22.3* expression is sensitive to *Ppa-die-1* and *Ppa-cog-1* perturbation, while *Ppa-gcy-22 .5* is largely independent of these canonical ASE laterality regulators. Both paralogs require the miRNA, *Ppa-miR-8345* , for repression in ASEL; loss of *Ppa-miR-8345* produces a complete ASEL-to-ASER conversion, in contrast to the hybrid ASEL/ASER states caused by *Ppa-die-1* or *Ppa-cog-1* mutation alone. These findings indicate *Ppa-miR-8345* acts through additional regulatory outputs beyond the *die-1* / *cog-1* feedback loop, revealing gene-by-gene rewiring of terminal differentiation programs.

**Figure 1. Transgenic promoter reporters and HCR-FISH show representative expression of P. pacificus ASER-specific Ppa-gcy genes:** The paralogs *Ppa-gcy-22 .3* and *Ppa-gcy-22 .5* mark ASER fate (green). The *Ppa-gcy-8.1* expression (magenta) in the AFD neurons represents the HCR-FISH staining control, while the ASEL-specific fate is marked by *Ppa-gcy-7.2* expression (red). (A) *Ppa-gcy-22 .3p::GFP* expression in the wild type is found only in the ASER neuron. (B-B') Reduction of *Ppa-* DIE-1 function results in 2xASER* expression of *Ppa-gcy-22 .3p::GFP* . (C-C') *Ppa-miR-8345* mutants only show the 2xASER *Ppa-gcy-22 .3p::GFP* misexpression. (D) A proposed model for the paralog-specific negative regulatory loop controlling *Ppa-gcy-22* paralog expression. (E) HCR-FISH shows *Ppa-gcy-22 .3* expression in the wild type is found only in the ASER neuron. (F) *Ppa-die-1* mutants have the 2xASER* *Ppa-gcy-22 .3* ectopic expression with a hybrid ASEL neuron. (G-G') *Ppa-miR-8345* mutants show 2xASER misexpression of *Ppa-gcy-22 .3* . (H-H') The loss of negative regulatory sites in the *Ppa-cog-1* 3' UTR (gain-of-function allele) results in the misexpression of *Ppa-gcy-22 .3* . (I) *Ppa-gcy-22 .5p::GFP* expression in the wild type is found only in the ASER neuron. (J) *Ppa-die-1* mutants show wildtype-like *Ppa-gcy-22 .5p::GFP* expression. (K) *Ppa-miR-8345* mutants result in the 2xASER *Ppa-gcy-22 .5p::GFP* misexpression. (L) The loss of negative regulatory sites in the *Ppa-cog-1* 3' UTR results in the 2xASER *Ppa-gcy-22 .5p::GFP* expression. (M) *Ppa-gcy-22 .5* expression in the wild type is found only in the ASER neuron. (N-N') A reduction-of-function mutation in *Ppa-die-1* does not significantly alter *Ppa-gcy-22 .5* expression. (O-O') *Ppa-miR-8345* mutants show only 2xASER *Ppa-gcy-22 .5* misexpression. (P-P') The loss of negative regulatory sites in the *Ppa-cog-1* 3' UTR does not affect *Ppa-gcy-22 .5* expression. Arrowheads indicate misexpression of the ASER marker in the ASEL neuron. The percentage refers to the animals with specific ASER expression pattern with the sample number indicated in the parentheses. Results in B, F-H, and J were previously reported (Castro *et al* , 2026) ^1^ . Scale bars: 5 μm.

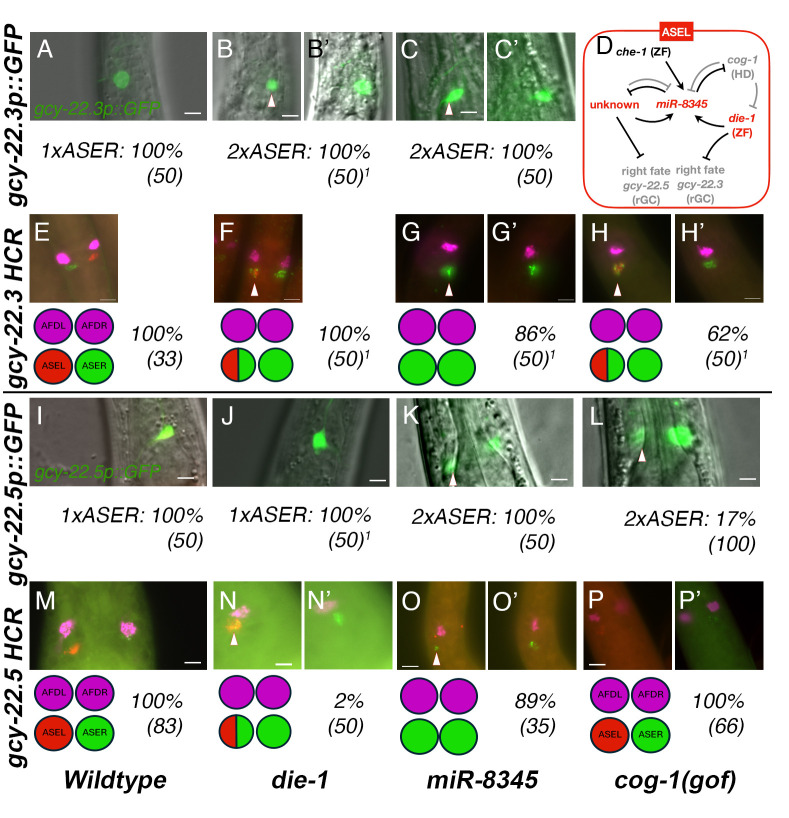



## Description

The establishment and maintenance of neuronal identity require activation and sustained expression of terminally differentiated genes such as neurotransmitters and taste receptors. For the specification of the ASE chemosensory neurons in *C. elegans* , the CHE-1/Glass-type zinc-finger protein is a paradigmatic terminal selector known to regulate a suite of 4 left-specific and 5 right-specific receptor-type guanylate cyclases ( *gcys* ) in the ASE neurons through direct binding of their regulatory regions (Yu et al. 1997; Johnston et al. 2005; Ortiz et al. 2006). In addition, three regulators- DIE-1 (zinc-finger), *lsy-6* (miRNA), COG-1 (homeodomain) - form a negative regulatory feedback loop to produce mutually exclusive expression of either *cog-1* or *die-1* in the ASEL and ASER neurons, respectively (Palmer et al. 2002; Chang et al. 2003; Johnston and Hobert 2003; Johnston et al. 2005). Thus, ASEL-specific ( *gcy-6 , gcy-7 )* as well as ASER-specific ( *gcy-5 , gcy-22 ) gcy* genes are controlled by the same genetic regulators (Johnston et al 2005). However, it remains unclear if such miRNA regulatory network abides by the same conserved principle to establish stable terminally differentiated receptor gene expression in other nematodes.

In the left/right asymmetric ASE gustatory neuron pair of the predatory, entomophilic nematode *Pristionchus pacificus* , the *Ppa-* CHE-1-dependent ASER-specific *Ppa-gcy-22* subfamily ( *gcy-22.1, gcy-22.2, gcy-22.3, gcy-22.4, gcy-22.5)* provides a comparative context to examine the role of the regulatory miRNA in establishing neuronal asymmetry. These five *Ppa-gcy-22* paralogs are distributed across 4 loci over 3 chromosomes (I, IV, X), with *Ppa-gcy-22 .3* *and Ppa-gcy-22 .5* located on Chromosomes IV and X, respectively. Notably, *Ppa-die-1 r* eduction-of-function mutants show a fully penetrant misexpression of the ASER marker *Ppa-gcy-22 .3* in the ASEL, while *Ppa-gcy-22 .5* expression remains wildtype-like in *Ppa-die-1 (csu225)* , indicating that detectable repression of *Ppa-gcy-22 .5* in ASEL does not require normal DIE-1 function under these assay conditions. (Castro et al 2026). Here we show that two ASER-expressed *Ppa-gcy-22* paralogs differ in their dependence on canonical ASE laterality regulators: *Ppa-gcy-22 .3* is sensitive to *Ppa-die-1* and *Ppa-cog-1* perturbation, whereas *Ppa-gcy-22 .5* is largely independent of those transcriptional regulators but remains dependent on *Ppa-miR-8345* for repression in ASEL. Understanding the roles of miRNA-mediated regulation can expose the veneer of genetic conservation to show paralog-specific branches of terminal differentiation programs.

To determine the extent of paralog-specific regulation in the ASE neurons, we used both transgenic reporters and HCR-FISH (Hybridization Chain Reaction *In situ* Fluorescent Hybridization) to characterize *Ppa-gcy-22 .3* *and Ppa-gcy-22 .5* expression in mutants comprising this miRNA regulatory loop. While the reporters allow us to assess promoter activity through likely conserved *Ppa-* CHE-1-dependent ASE-motifs in the *cis* -regulatory regions of the *gcy* genes (Etchberger et al 2007), the recent utilization of HCR-FISH permits the simultaneous monitoring of multiple mRNA transcripts to determine if the ASEL neuron expresses a stable hybrid state (a *Ppa-gcy-22* paralog and *Ppa-gcy-7.2* ) or if the ASEL neuron is transformed completely into the ASER fate (expresses only the *gcy-22* paralogs). We use “2xASER” to denote animals in which both ASE neurons express ASER markers and lack detectable ASEL-specific *Ppa-gcy-7.2* expression. “2xASER*” is used to denote ectopic ASER-marker expression in ASEL neurons while ASEL-specific *Ppa-gcy-7.2* expression persists, indicating a hybrid ASEL/ASER state.

As reported previously, *Ppa-gcy-22 .3p::GFP* and *Ppa-gcy-22 .3* steady-state transcript expression patterns show mis-expression in the ASEL in reduction-of-function *Ppa-die-1 (csu225)* mutants (Castro et al 2026), whereas *Ppa-gcy-22 .5p::GFP* and *Ppa-gcy-22 .5* transcript expression in *Ppa-die-1 (csu225)* remain wildtype-like and restricted to the ASER ( Fig. 1 B, F, J, N). With the exception of a single animal misexpressing *Ppa-gcy-22 .5* in both ASE neurons (2% 2xASER*, n=50), we found predominantly wild-type *Ppa-gcy-22 .5* expression patterns in *Ppa-die-1* mutants ( Fig. 1N ). By contrast, GFP reporters and FISH detected misexpression of both *Ppa-gcy-22 .3* (86% 2xASER) and *Ppa-gcy-22 .5* (89% 2xASER, n=35) in both ASE neurons in *Ppa-miR-8345(csu259)* mutants, without any ASEL-specific *Ppa-gcy-7.2* expression ( Fig. 1 C, G, K, O). Despite functional similarity, *Ppa-miR-8345* does not appear to be an ortholog of *C. elegans lsy-6* , because its precursor sequence, predicted hairpin structure, and genomic location differ substantially (Castro et al, 2026). Finally, while *Ppa-gcy-22 .3* transcript expression in the *Ppa-cog-1* gain-of-function allele shows the 2xASER* phenotype with hybrid ASEL (62%)( Fig. 1H- H'), *Ppa-gcy-22 .5* expression was wildtype-like and detectable only in the ASER (100%, n=66)( Fig. 1P- P'). Interestingly, in the *Ppa-cog-1 (csu255)* gain-of-function allele, *Ppa-gcy-22 .5p::GFP* expression occasionally exhibited the 2xASER phenotype (17%, n=100)( Fig. 1L ), although this effect was not detected at the endogenous transcript level. Taken together, these results indicate that loss of *Ppa-miR-8345* produces a more complete molecular conversion of ASEL toward ASER identity than either *Ppa-die-1* reduction of function or *Ppa-cog-1* derepression alone. In *Ppa-die-1* and *Ppa-cog-1* mutant animals, *Ppa-gcy-22 .3* can be ectopically expressed in ASEL while ASEL-specific *Ppa-gcy-7.2* expression persists, consistent with a hybrid ASEL/ASER state. By contrast, *Ppa-miR-8345* mutants ectopically express both *Ppa-gcy-22 .3* and *Ppa-gcy-22 .5* in both ASE neurons and lack detectable ASEL-specific *Ppa-gcy-7.2* expression. These observations suggest that *Ppa-miR-8345* controls additional regulatory output(s), beyond the canonical *die-1/cog-1* bistable loop that are required to suppress ASER-specific *gcy* paralogs in ASEL. The differential response of *Ppa-gcy-22 .3* and *Ppa-gcy-22 .5* further suggests that activation of ASEL-specific *Ppa-gcy-7.2* and repression of ASER-specific *Ppa-gcy-22* paralogs are genetically separable outputs, rather than inseparable consequences of a single binary fate switch.

Whereas the asymmetrically expressed ASE *gcy* genes undergo a hybrid precursor state in late embryogenesis and early larval stages in *C. elegans* , transcripts of the ASEL- and ASER-specific *gcy* genes have not been detected to co-localize in the same ASE neuron in *P. pacificus (* Johnston et al 2005, Castro et al 2026). *P. pacificus gcy-22.3* and *Ppa-gcy-7.2* however, do exhibit precursor hybrid states in the AFD thermosensory neurons during late embryogenesis that resolve into a *Ppa-gcy-8.1* -only state by the J1 early larval stage. Thus, hybrid fates in the ASE neurons do not represent delayed paedomorphic phenotypes but rather bona fide changes in post-mitotic terminal cell fates. It is unclear which other target of *Ppa-miR-8345* mediates the suppression of *Ppa-gcy-22 .5* in the ASEL neuron. Our finding shows that regulation of terminal selector outputs and asymmetric effector genes may be rewired on a gene-by-gene basis, rather than acquired as a single coordinated regulatory module.

## Methods

*P. pacificus* and other nematode strains were maintained at ~20°C on NGM plates seeded with *E. coli* OP50 . For reporter analyses, we crossed *Ppa-gcy-22 .3p::gfp* and *Ppa-gcy-22 .5p::gfp* males with *Ppa-miR-8345(csu259)* and *Ppa-cog-1 (csu255)* mutant hermaphrodites and identified transgenic homozygous mutant progeny by sequencing PCR products. For FISH, we performed third-generation HCR v3.0 with split-initiator probe pairs using three differently conjugated fluorophores (B2, B4, B5)(Molecular Instruments, Los Angeles, CA) as previously described (Castro et al. 2026). Although the transcript levels for *Ppa-gcy-22 .5* (PPA03763) on www.pristionchus.org appear only in some but not all expression datasets (compare stage-specific expression in Baskaran et al, 2015 with Han and Lo, 2022), we were able to consistently observe HCR-FISH signals for *Ppa-gcy-22 .5,* albeit at distinctively lower level than *Ppa-gcy-22 .3* (PPA04464). We ordered the probe sets as DNA oligo pools at 50 pmol (IDT, San Diego, CA) and used a 10x higher concentration of probes than previously published (20 pmol) (Ramadan and Hobert 2024). Because no obvious stage-dependent differences were observed among post-embryonic stages, animals from J2 through adult stages were pooled for phenotype scoring (J2, J3, J4, adult). For *Ppa-gcy-22 .5* , a new round of HCR-FISH was performed a year later (wild type, *Ppa-die-1 , Ppa-cog-1* ) and combined with previously published results.

**Table:** 

**strain name**	**genotype**	**source**
PS312	Wildtype	
RLH334	* csuEx90 [ Ppa-gcy-22 .3p::gfp; Ppa-egl-20p::rfp] *	Castro et al, 2026
RLH333	* csuEx90 [ Ppa-gcy-22 .3p::gfp; Ppa-egl-20p::rfp]; Ppa-die-1 (csu225) *	Castro et al, 2026
RLH405	* csuEx90 [ Ppa-gcy-22 .3p::gfp; Ppa-egl-20p::rfp]; Ppa-miR-8345 (csu259) *	this study
RLH378	* csuEx105 [ Ppa-gcy-22 .5p::gfp; Ppa-egl-20p::rfp] *	Castro et al, 2026
RLH346	* csuEx105 [ Ppa-gcy-22 .5p::gfp; Ppa-egl-20p::rfp]; Ppa-die-1 (csu225) *	Castro et al, 2026
RLH397	* csuEx105 [ Ppa-gcy-22 .5p::gfp; Ppa-egl-20p::rfp]; Ppa-miR-8345 (csu259) *	this study
RLH387	* csuEx105 [ Ppa-gcy-22 .5p::gfp; Ppa-egl-20p::rfp]; Ppa-cog-1 (csu255) *	this study
